# A Quantitative Comparison of Multispectral Refraction Topography and Autorefractometer in Young Adults

**DOI:** 10.3389/fmed.2021.715640

**Published:** 2021-09-13

**Authors:** Yunru Liao, Zhenlan Yang, Zijing Li, Rui Zeng, Jing Wang, Yichi Zhang, Yuqing Lan

**Affiliations:** ^1^Department of Ophthalmology, Sun Yat-sen Memorial Hospital, Sun Yat-sen University, Guangzhou, China; ^2^Department of Glaucoma, Zhongshan Ophthalmic Center, Sun Yat-sen University, Guangzhou, China

**Keywords:** consistency, multispectral refraction topography, autorefractometer, refraction, multispectral imaging

## Abstract

**Purpose:** Purpose of this study is to evaluate the measuring consistency of central refraction between multispectral refraction topography (MRT) and autorefractometry.

**Methods:** This was a descriptive cross-sectional study including subjects in Sun Yat-sen Memorial Hospital from September 1, 2020, to December 31, 2020, ages 20 to 35 years with a best corrected visual acuity of 20/20 or better. All patients underwent cycloplegia, and the refractive status was estimated with autorefractometer, experienced optometrist and MRT. We analyzed the central refraction of the autorefractometer and MRT. The repeatability and reproducibility of values measured using both devices were evaluated using intraclass correlation coefficients (ICCs).

**Results:** A total of 145 subjects ages 20 to 35 (290 eyes) were enrolled. The mean central refraction of the autorefractometer was −4.69 ± 2.64 diopters (D) (range −9.50 to +4.75 D), while the mean central refraction of MRT was −4.49 ± 2.61 diopters (D) (range −8.79 to +5.02 D). Pearson correlation analysis revealed a high correlation between the two devices. The intraclass correlation coefficient (ICC) also showed high agreement. The intrarater and interrater ICC values of central refraction were more than 0.90 in both devices and conditions. At the same time, the mean central refraction of experienced optometrist was −4.74 ± 2.66 diopters (D) (range −9.50 to +4.75D). The intra-class correlation coefficient of central refraction measured by MRT and subjective refraction was 0.939.

**Conclusions:** Results revealed that autorefractometry, experienced optometrist and MRT show high agreement in measuring central refraction. MRT could provide a potential objective method to assess peripheral refraction.

## Introduction

Myopia is by far the most common refractive error and a dominant reason of visual impairment globally (1), with a prevalent rate of 10–30% of adults in many countries and 80–90% of young people in some parts of East and South-East Asia (2, 3). Myopia of −6.00 diopters (D) or more severe is called high myopia and is often causes visual impairment due to complications such as posterior uveioma, choroidal new vascularization, retinal detachment and so on (4). Reducing the high myopia incidence rate and improving the quality of life are the goal of prevention and treatment of myopia.

Animal experiments have provided details about myopia: hyperopic defocus increases axial elongation, while myopic defocus decreases axial elongation (4–11). Retinal peripheral visual signals, which are basically the sum of regions, can contral central refractive development independent of central visual experience. The effectiveness of optical defocus in changing axial elongation depends on retinal defocus degree (12, 13). However, there are generally four methods to evaluate eccentric refractive errors (14): subjective eccentric refraction (15), wavefront measurements with an aHS sensor (16), streak retinoscopy (17), and photo refraction with a power refractor (14). However, these methods can only detect a small area of the retina and cannot accurately detect the peripheral defocus of each region of the retina. Further, the process has high requirements for patient cooperation, and it is cumbersome, time-consuming, and difficult to adapt to clinical practice (18, 19).

MRT is a new instrument using multispectral imaging technology (MSI). MSI is an emerging technology based on imaging and spectroscopy. It is the result of remote sensing technology as a kind of analysis tool and can obtain information on the measured target simultaneously from the spectral and spatial dimensions. MRT can detect the refraction of each part of the retina within a range of 30° at the posterior pole of the retina, especially the refraction of the fovea of the macula.

The usage of different technologies in the MRT and other devices above talking about may result in differences in measurements. Because treatment centers use different topographic devices, differences in such measurements might lead to differences between diagnostic or treatment centers.

Therefore, we evaluated agreement between MRT and autorefractometer to see whether they could be interchangeable used or not. The data offered by each device should be maintained consistent at different measurements so that results can be used in research. Hence, we evaluated the repeatability of the devices' measurements to decide their effectiveness and availability.

## Methods

The present research was a descriptive cross-sectional study, and it was conducted in accordance with the tenets of the 1964 Declaration of Helsinki and its later amendments or comparable ethical standards. Local ethical approval (SYSEC-KY-KS-2021-061) was obtained from the Ethics Committee of Sun Yat-sen Memorial Hospital at Sun Yat-sen University, Guangzhou, China. The medical records of consecutive patients in Sun Yat-sen Memorial Hospital from September 1, 2020 to December 31, 2020 were reviewed. The inclusion criteria were as follows: (1) subjects ages 20 to 35 years, (2) subjects with a best corrected visual acuity of 20/20 or better, (3) subjects with MRT results, (4) subjects with the refraction results of autorefractometer and experienced optometrists. Exclusion criteria were as follows: (1) intraocular pressure higher than 21 mmHg, (2) a history of ocular diseases or previous ocular surgery that may influence refraction or axial length, such as corneal and lens diseases, and (3) a history of corneal contact lens, such as orthokeratology.

The refractive errors of all eyes were measured by both an autorefractometer (AR-360A, NIDEK Co., Ltd, Japan), an experienced optometrist, and MRT (version 1.0.5T05C; Thondar, Inc.) Thirty minutes before examination, a cycloplegic agent (one drop of 0.5% tropicamide along with 0.5%) phenylephrine hydrochloride (Sinqi Pharmaceutical Co. Ltd., Shenyang, China) was applied 3 times (with 5 min between each application). The mean of three consecutive autorefraction readings was collected as the refractive error value measured by the autorefractometer. MRT measurements use MSI technology for central refraction measurements. To compare the two devices, the central refraction values from the two devices were analyzed. All examinations were performed by the same experienced doctor.

Statistical analysis was performed using SPSS (version 23). Intra-class correlation coefficient (ICC) and repeated measurement analysis of variance (ANOVA) were used to evaluate the repeatability of the equipment. Pearson's correlation coefficient, paired *t*-test, and Bland-Altman plots were used to compare the two devices. A value of *P* < 0.05 was taken to indicate statistical significance.

## Results

This study enrolled 290 eyes of 145 subjects. Baseline characteristics of the subjects are shown in Table 1.

**Table 1:** Baseline characteristics of the participants.

**Characteristics**			**Value**
Subjects (eye)			145 (290)
Sex	Female		105
	Male		40
Age	Mean		26.23(SD2.62)
	Range		20–35
Emmetropia			3
Ametropia	Myopia	Low(−0.25~−3.00D)	61
		Medium(−3.25~−6.00D)	121
		High(< −6.00D)	93
	Hyperopia	Low(+0.25~+3.00D)	10
		Medium(+3.25~+5.00D)	2
		High(>+5.00D)	0

*SD, standard deviation*.

**Figure 1:**
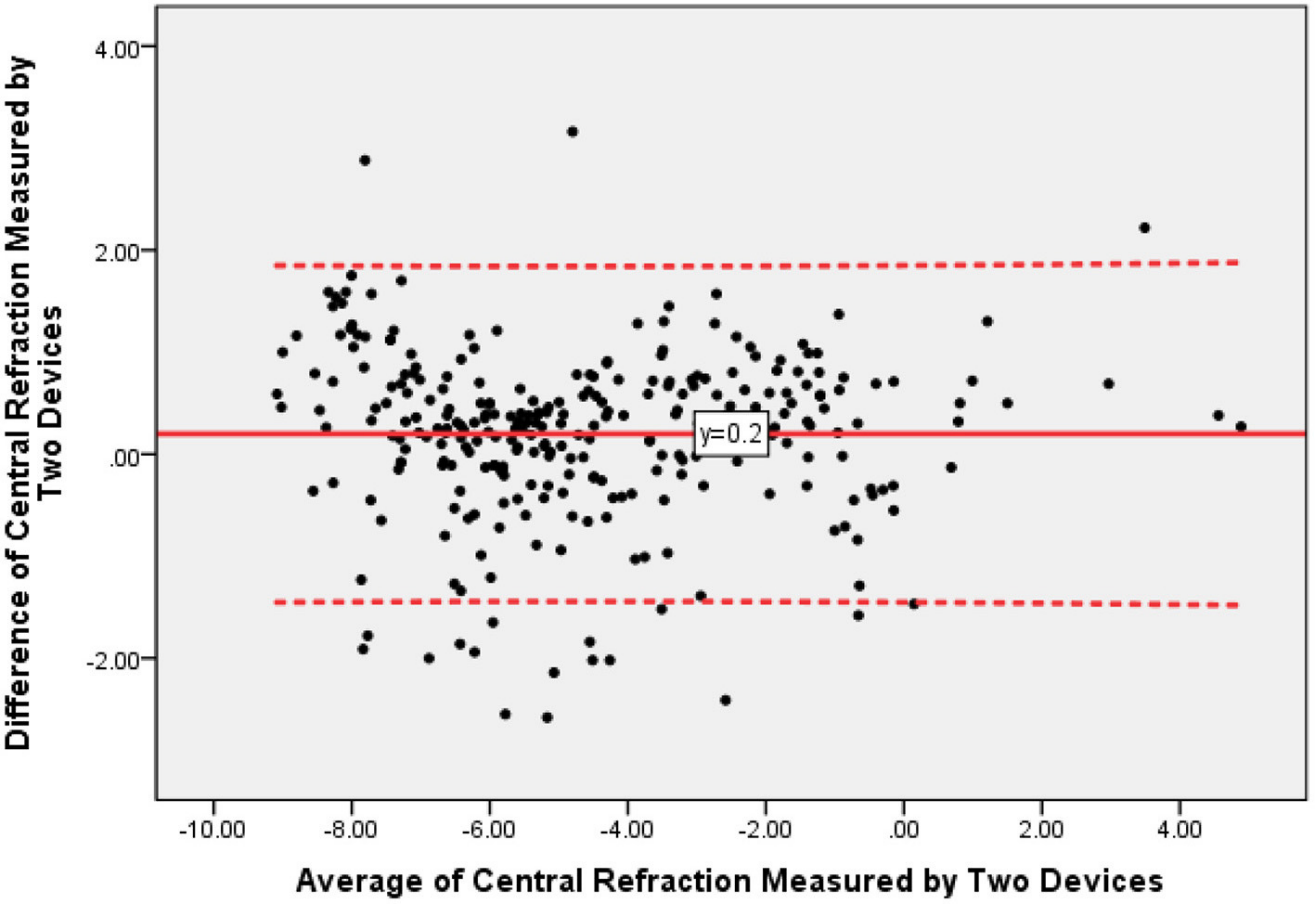
The Bland-Altman plots of central refraction measured by multispectral refraction topography (MRT) and autorefractometer.

All 290 eyes were measured by one technician, and all values were collected for further analysis. The mean central refraction measured by an autorefractometer was −4.69 ± 2.64 diopters (D) (range −9.50 to +4.75 D), while the mean central refraction measured by MRT was −4.49 ± 2.61 diopters (D) (range −8.79 to +5.02 D). Pearson correlation analysis revealed a high correlation between MRT and autorefractometer results (*R* = 0.950, *P* < 0.001). Figure 1 showed the difference values of central refraction between MRT and autorefractometer vs. the average of these two results. The mean difference value was 0.20 D while the 95% confidence interval was −1.43 to +1.83 D.

**Figure 2:**
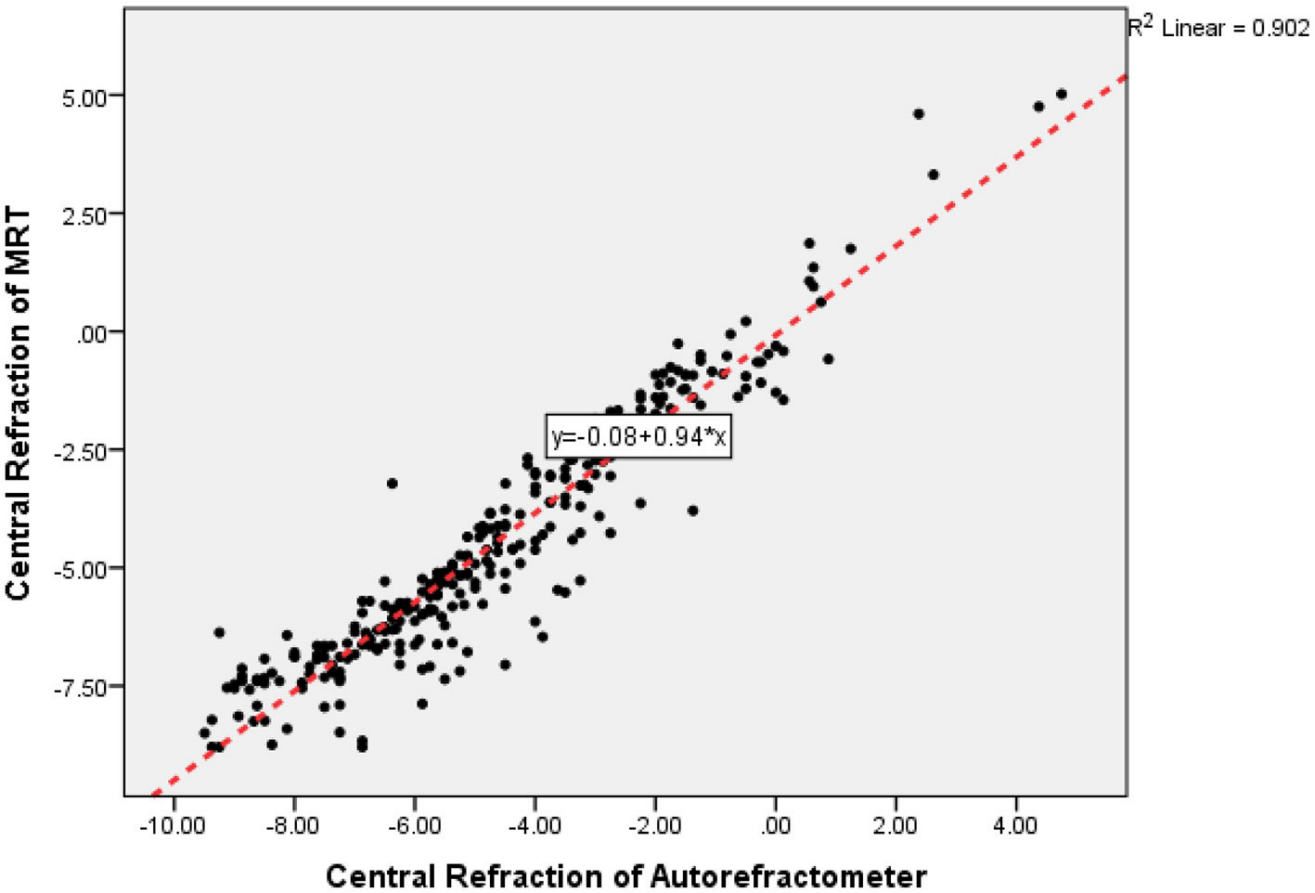
The correlations of central refraction between multispectral refraction topography (MRT) and autorefractometer.

The correlations of central refraction of the two devices were shown in Figure 2 (*R* = 0.947, *P* < 0.001).

Tables 2, 3 showed the description and intraclass correlation coefficient (ICC) for central refraction of autorefractometer and multispectral refraction topography. The intrarater and interrater ICC values were 0.947 and 0.973, respectively.

**Table 2:** Measured Values and Paired *T*-test Results by Autorefractometer and Multispectral Refraction Topography (MRT).

	**Mean**	**Standard deviation**	**Range**	***p*** **-value(2-tailed)** **(paired samples test)**
Central refraction of MRT	−4.49D	2.61	−8.79D to +5.02D	0.000
Central refraction of autorefractometer	−4.69D	2.64	−9.50D to +4.75D	

**Table 3:** Intraclass Correlation Coefficient.

	**Intraclass**	**95% confidence interval**		**F test with true value 0**	
	**Correlation^b^**	**Lower bound**	**Upper bound**	**Value**	**Sig**
Single measures	0.947^a^	0.931	0.959	38.740	0.000
Average measures	0.973	0.964	0.979	38.740	0.000

*Two-way random effects model where both individual effects and measure effects are random*.

a *The estimator is the same whether the interaction effect is present or not*.

b *Type An intraclass correlation coefficients using an absolute agreement definition*.

**Figure 3:**
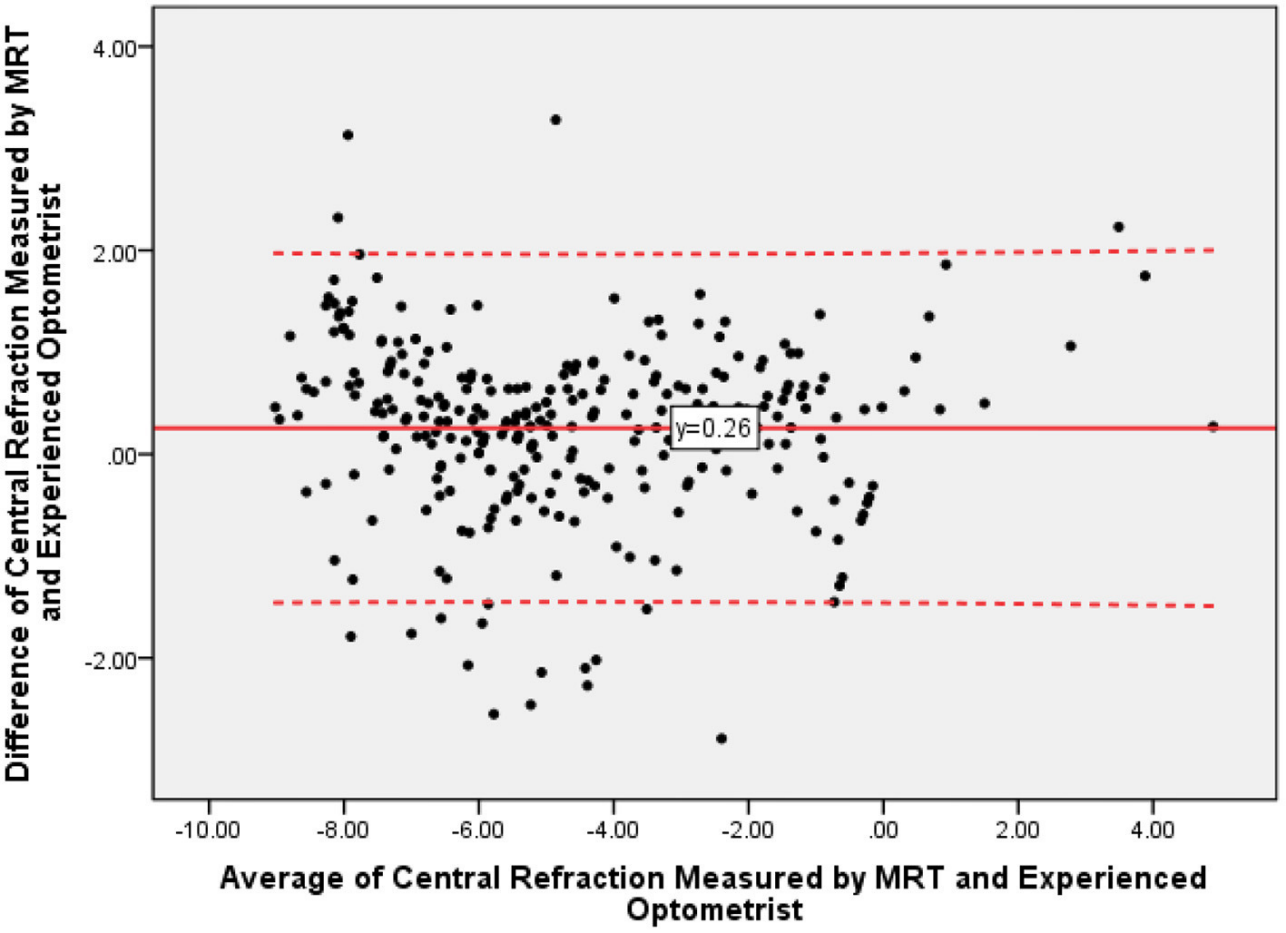
The Bland-Altman plots of central refraction obtained by MRT and experienced optometrist.

**Figure 4:**
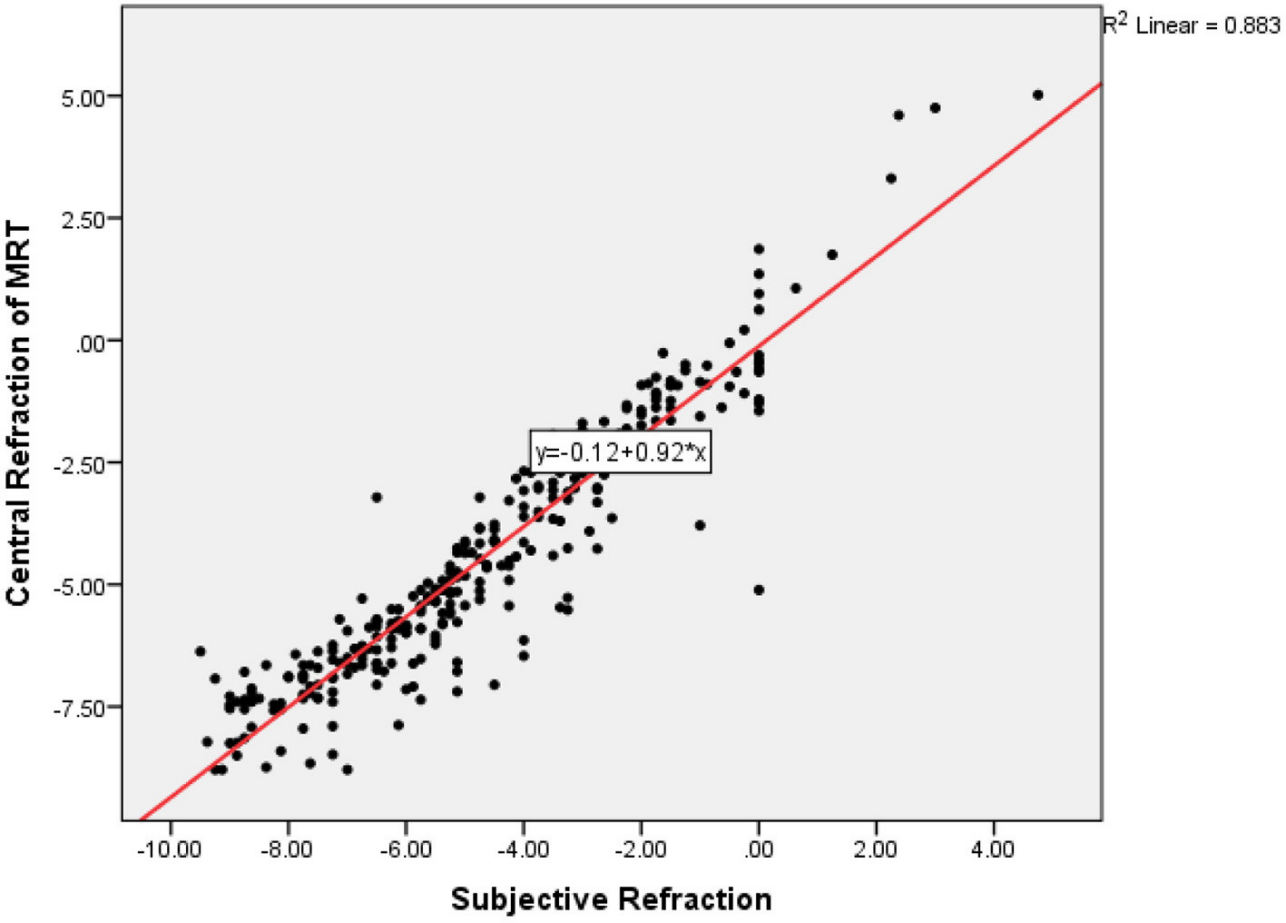
The correlations of central refraction obtained by MRT and experienced optometrist.

Then we analyzed the result of MRT measurement and subjective refraction. The mean subjective refraction was −4.74 ± 2.66D, its range was −9.50 to +4.75D. The mean of the difference between the central refraction obtained by the MRT measurement and the experienced optometrist was 0.26 ± 0.87D, and its 95% confidence interval was −1.44 to +1.95 (Figure 3). The intra-class correlation coefficient of central refraction measured by MRT and subjective refraction was 0.939 (Figure 4, *P* < 0.001).

## Discussion

The study demonstrates that the central refraction obtained by autorefractometer devices, experienced optometrists, and MRT shows high repeatability and reproducibility. Our results indicate that MRT is a valid and safe method for measuring central refraction error in healthy eyes, particularly for mild myopia. Furthermore, the values showed a high correlation between the two devices. Comparing with autorefractometer or experienced optometrist, measured values by MRT showed a statistically significant shift toward hyperopia. This difference is about 0.20 D (comparing with autorefractometer) to 0.26D (comparing with subjective refraction). It suggests that the accommodation reflex may still have played a role in these participants. We consider the MRT test a more powerful tool to measure the full hyperopic refractive error.

To date, current research suggests that the surrounding area of the eye also plays an important role in controlling the growth of the eye and the development of refractive errors. Peripheral hyperopic defocus of the retina is one of the causes of myopia. If the defocus degree of the retina, especially the peripheral defocus, can be measured effectively and accurately, it will be helpful in preventing myopia. The reason for using objective methods to test peripheral defocus is to try to find the “gold standard” compared with how conventional subjective refraction is used. An ideal screening test should be perfect in specificity, sensitivity, and positive predictive value, but we still can't find any screening method that achieves this level of accuracy. The current methods used to measure peripheral refraction are more difficult to evaluate due to poor retinal image quality, optical aberration and low retinal resolution, which may result in insufficient retinal image sampling (14).

MRT is a new instrument using MSI that can accurately measure the refraction of each part of the retina, and in a sense, it can replace the role of autorefractometer measurement. However, because the device is a new technology, its accuracy must be compared with the traditional gold standard. Autorefractometers have been used for several decades. They are used in optometric practice all around the world, primarily as a starting point for ophthalmologists or optometrists to assess subjective refraction (20). Autorefractometers are currently the gold standard for testing refration of the central retina. In young adults, most of the time, we would use cycloplegic refraction to detect refractive errors, which is also the gold standard now. Hence, if the central refraction measured by MRT and autorefractometers is consistent in cycloplegic cases, we can assume that the MRT accurately reflects the level of refraction of each part of the retina. MRT is a rapid, accurate, and noninvasive refractometer, and it has excellent specificity and sensitivity. The data of our experiment, under cycloplegic conditions, confirmed that compared with traditional refractometers, MRT can accurately measure central refraction, and the results are closely related to those of autorefractometers. There was no significant difference between the two devices (Pearson correlation coefficient test, *P* < 0.001). To our knowledge, this is the first report of the consistency between MRT and the autorefractometer.

Nevertheless, our experiment has limitations. The subjects of this experiment were Asian individuals ages 20–35 who were treated at Sun Yat-sen Memorial Hospital, and no other ethnic groups were involved. Therefore, further experiments are needed to prove whether this instrument is suitable for other populations. The results included in our study were mostly myopic patients and a few hyperopic patients; there were no patients with high hyperopia, because the greatest proportion of myopic patients in China. In the future, we will collect the results of hyperopic patients, especially those with high hyperopia, to clarify the accuracy of the instrument.

In conclusion, results revealed that autorefractometry and MRT show high agreement in measuring central refraction. MRT can accurately reflect the refraction of the retina. It can therefore be used as a potential objective method to measure peripheral defocus of the retina.

## Data Availability Statement

The raw data supporting the conclusions of this article will be made available by the authors, without undue reservation.

## Ethics Statement

The studies involving human participants were reviewed and approved by the Ethics Committee of Sun Yat-sen Memorial Hospital at Sun Yat-sen University. Written informed consent for participation was not required for this study in accordance with the national legislation and the institutional requirements.

## Author Contributions

YLi, ZY, and YZ contributed to conception and design of the study. ZY, RZ, and JW organized the database. YLi and ZL performed the statistical analysis. YLi wrote the first draft of the manuscript. RZ, ZY, ZL, and YZ wrote sections of the manuscript. All authors contributed to manuscript revision, read, and approved the submitted version.

## Funding

This study was funded by the National Natural Science Foundation of China (Grant No: 81700833) and the Sun Yat-sen Clinical Research Cultivation Program of Sun Yat-sen Memorial Hospital, Sun Yat-sen University (No. SYS-C-201705).

## Conflict of Interest

The authors declare that the research was conducted in the absence of any commercial or financial relationships that could be construed as a potential conflict of interest.

## Publisher's Note

All claims expressed in this article are solely those of the authors and do not necessarily represent those of their affiliated organizations, or those of the publisher, the editors and the reviewers. Any product that may be evaluated in this article, or claim that may be made by its manufacturer, is not guaranteed or endorsed by the publisher.
